# Gender differences in the use of insecticide-treated nets after a universal free distribution campaign in Kano State, Nigeria: post-campaign survey results

**DOI:** 10.1186/1475-2875-12-119

**Published:** 2013-04-10

**Authors:** Ashley E Garley, Elizabeth Ivanovich, Erin Eckert, Svetlana Negroustoueva, Yazoume Ye

**Affiliations:** 1 ICF International, 11785 Beltsville Drive, Suite 300, Calverton, MD 20705, USA; 2 The US President’s Malaria Initiative/USAID, Washington, DC, USA; 3 Independent Consultant, Kinshasa, Democratic Republic of Congo

**Keywords:** Long-lasting insecticidal net, Insecticide-treated net, Net use, Gender, Universal coverage, Health disparities, Survey, Free distribution campaign, Kano State, Nigeria

## Abstract

**Background:**

Recent expansion in insecticide-treated net (ITN) distribution strategies range from targeting pregnant women and children under five and distributing ITN at antenatal care and immunization programmes, to providing free distribution campaigns to cover an entire population. These changes in strategy raise issues of disparities, such as equity of access and equality in ITN use among different groups, including females and males. Analysis is needed to assess the effects of gender on uptake of key malaria control interventions. A recent post-universal free ITN distribution campaign survey in Kano State, Nigeria offered an opportunity to look at gender effects on ITN use.

**Methods:**

A post-campaign survey was conducted three to five months after the campaign in Kano State, Nigeria from 19 October to 4 November, 2009, on a random sample of 4,602 individuals. The survey was carried out using a questionnaire adapted from the Malaria Indicator Survey. Using binary logistic regression, controlling for several covariates, the authors assessed gender effects on ITN use among individuals living in households with at least one ITN.

**Results:**

The survey showed that household ITN ownership increased more than 10-fold, from 6% before to 71% after the campaign. There was no significant difference between the proportion of females and males living in households with at least one ITN. However, a higher percentage of females used ITNs compared to males (57.2% *vs* 48.8%). After controlling for several covariates, females remained more likely to use ITNs compared to males (OR: 1.5, 95% CI: 1.3-1.7). Adolescent boys remained the least likely group to use an ITN.

**Conclusions:**

This study reveals gender disparity in ITN use, with males less likely to use ITNs particularly among ages 15–25 years. The uptake of the intervention among the most at-risk group (females) is higher than males, which may be reflective of earlier strategies for malaria interventions. Further research is needed to identify whether gender disparities in ITN use are related to traditional targeting of pregnant women and children with malaria interventions; however, results provide evidence to design gender-sensitive messaging for universal ITN distribution campaigns to ensure that males benefit equally from such communications and activities.

## Background

Universal coverage through free insecticide-treated net (ITN) distribution campaigns is the current agreed-upon strategy to improve coverage in ownership of ITNs; however, traditional strategies such as social marketing and free distribution through antenatal care (ANC) and immunization campaigns are still recommended to reach pregnant women and children under five years. This universal coverage strategy has been facilitated by an increase in funding levels during recent years that have led to improved access to ITNs by allowing country programmes to provide the entire population with ITNs instead of limiting nets to high-risk groups [1]. Today, countries are rolling out these campaigns in an effort to achieve universal coverage, which is defined as one net per two household members, by 2015 [2]. Directed to the entire population, particularly in high-transmission settings, free ITN distribution campaigns have the advantage of rapidly achieving high community-level coverage, benefiting all community members rather than just those who own nets [3]. As shown by a number of studies in various settings, this strategy has the potential to achieve equity in ownership of mosquito nets and their use [4-7]. However, the level of its achievement depends largely on being context specific, i.e. using effective distribution strategies that address specific contextual determinants, such as gender.

Gender roles, norms, cultural practices, and behaviours can strongly influence disease prevention, care seeking, and access to treatment. These influences highlight the importance of addressing gender and other social determinants of health in policies and interventions to achieve gender equal returns from health programmes. Although men and women are both affected by malaria, social and biological factors contribute to the different impact malaria has on each. For instance, socially determined gender norms mean that women most often carry the extra burden of caring for sick family members, so malaria would affect their ability to work and provide care for young children and other members of the family. Women are also more susceptible to developing malaria due to a decreased immunity during pregnancy and an inequitable economic status that often results in delays to access health interventions [8].

On the other hand, men are less likely to use ITNs, which are traditionally distributed to pregnant women and children. In many cultures, men do not use health care routinely, leading to delays in seeking medical care when needed and, subsequently, omission of their cases in medical records. Moreover, in many malaria-endemic areas, certain gender-specific occupations may increase exposure to malaria vectors: men are more vulnerable to contracting malaria through occupational exposure (e.g. working in gold mines or forest logging and working at night) [9,10]. Men with untreated malaria infections are potential reservoirs for malaria transmission; therefore, it is essential to prevent all cases among both women and men.

Close attention needs to be paid to how inequalities facilitate the spread of disease, affecting the ability of both genders to access health care and other services equitably and in a manner that does not exacerbate gender inequalities that exist in society. Donors, including the Global Fund to Fight AIDS, Tuberculosis and Malaria and the President’s Malaria Initiative (PMI), assert that gender inequalities are a strong driver of malaria and that success in reducing malaria-related deaths can only be achieved through gender-sensitive programming [10,11]. Therefore, as countries strive for universal ITN ownership and use, it is important to assess gender dimensions to better inform programme implementation. However, there is limited evidence on the effects of gender, as a contextual and social determinant, on ITN uptake and its subsequent use, particularly after a free distribution campaign. Using data from the Kano post-campaign survey [7,12], the authors investigated the outcomes of the free mass ITN distribution campaign on ITN uptake and use through a gender lens, using sex-disaggregated data.

## Methods

### Study population

The post-campaign evaluation survey took place at the end of 2009 in Kano State, in the northwest region of Nigeria, and included 24 local government areas (LGA), which were covered by two independent campaign waves. During Wave 1, which took place in May 2009, the campaign covered 21 LGAs, while Wave 2 (July 2009) covered 23 LGAs.

This study was a cross-sectional household survey with a stratified two-stage cluster sampling design. The strata were the two areas covered in the different campaign waves and each stratum was considered a survey domain. The size of the sample was estimated based on confidence intervals (alpha-error) of 95%, statistical power (1-beta-error) of 80%, and a design effect of 1.75. It was assumed an anticipated nonresponse of 5% and an average household size of 5 people. Children under 5 constituted an estimated 20% of the population, while 3.5% of the female population was currently pregnant.

Stage one included the selection of clusters using systematic sampling with probability proportionate to size (PPS). Stage two included the selection of households by field team mapping small villages, randomly selecting 17 households and using the same household definition used in the distribution campaign, which was "a wife with her direct dependents". A compound was divided into several households depending on the number of wives and the husband was assigned to the first wife's household. Larger communities (more than 120 compounds) used the equal size section-approach.

Clusters were defined as urban or rural, based on their categorization in the 2006 census, not their urban/rural stratification. A sample of 30 clusters, with an estimated 17 households per stratum, resulted in a total sample of 1,020 households in the campaign area, including 4,602 individuals. Data collection took place from 19 October to 4 November, 2009 and involved a questionnaire adapted from the Malaria Indicator Survey [13]. The ITN was first identified before asking a standard question (‘Was this net slept under by any person last night?’) to the respondent for determining ITN use in the household. Further details on the survey methodology are presented elsewhere [7].

### Analysis

The total sample included 4,602 participants; however, only individuals (3,056) living in households with at least one ITN and sleeping in the households the night before the survey visit (de facto population) were included in the analysis. It should be noted that in Wave 1, the official definition of household used was from the 2008 NDHS. This definition stated that a household was “a person or a group of persons, related or unrelated, who live together and share common cooking and eating arrangements”. However, during the Wave 2 campaign, each wife, with her direct dependents, was defined as a household in polygamous households. For the purpose of this analysis, the authors used the NDHS’ definition of household and adapted the data from Wave 2.

The determination of net ownership consisted of computing two key malaria indicators: (a) the percentage of individuals living in households with at least one ITN and (b) the percentage of individuals using an ITN in households with at least one ITN, by sex and background characteristics.

Binary response logistic regression was used to assess the effect of sex on ITN use among individuals living in households with at least one ITN. The model controlled for covariates reported by other studies to be associated with net use, including household wealth quintiles, campaign waves (Wave 1– May 2009 and Wave 2 – July 2009), age of individual, place of residence, education of the head of household, polygamous households, and ratio of ITNs to household members. The campaign wave covariate was chosen to determine whether messages from campaign waves varied by gender and affected ITN use. Age was selected since different age groups have dissimilar needs for or beliefs about using ITNs. Place of residence was chosen to see if the universal campaign messages were accessible in both rural and urban areas. Since northern Nigeria is comprised of a predominantly traditional, conservative population where the head of the household is often the decision-maker, the education level of the head of household was considered. The polygamous household variable was also measured since the ITN distribution strategy during the campaign considered every wife as a household unit; however, in the analysis, the official definition of a household was used in order to be comparable to those used as demographic health survey indicators. Finally, previous studies have shown that more nets in a household lead to better net usage, thus analysing the ratio of ITNs to household members was essential. The statistical significance was tested at the 95% level.

## Results

### Population characteristics

The study sample included 4,602 individuals, 51% of which were female and 49% of which were male; the majority of respondents were between 15 and 49 years old. Sixty-six percent of those surveyed lived in rural areas with an even distribution of females and males. The majority of the study sample (65%) reported having no formal education, while 19% completed primary level and 16% completed secondary level.

### Household ownership of ITNs

Of the 4,602 individuals surveyed in the post-campaign survey, 66% lived in households that owned at least one ITN, with no difference in ownership between females (67%) and males (66%). Wave 2 (July 2009) revealed better results than Wave 1 (May 2009), overall. There was no difference by sex in the proportion of individuals living in households that owned at least one ITN in either rural or urban areas. Individuals belonging to households in the second and fourth wealth quintiles were the most likely to own at least one ITN (71% and 70%, respectively), followed by the middle (68%) and lowest (64%) quintiles. The highest wealth quintile had the lowest (61%) ITN ownership (Table 1).

**Table 1:** Percentage of individuals living in households with ITNs, by gender and background characteristics

**Background characteristics**	**Female**		**Male**		**Total**	
	**Percentage**	**N**	**Percentage**	**n**	**Percentage**	**N**
**Total**	**66.7**	**2,342**	**66.1**	**2,260**	**66.4**	**4,602**
**Campaign waves**						
Wave 1	63.8	1,169	63.3	1,175	63.6	2,344
Wave 2	69.6	1,173	69.1	1,085	69.4	2,258
**Place of residence**						
Urban	69.5	791	68.5	774	69.0	1,565
Rural	65.3	1,551	64.9	1,486	65.1	3,037
**Wealth quintiles**						
Lowest	62.3	453	65.7	364	64.0	817
Second	70.0	466	71.4	398	70.7	864
Middle	69.3	452	66.2	494	67.7	946
Fourth	70.6	472	69.0	464	69.8	936
Highest	61.7	499	60.0	540	60.9	1,039

### ITN use

Overall, ITN use among individuals living in households with at least one ITN was 53%; however, there was a significant difference in use between females and males (57% *vs* 53%, p<0000.1). Consistently, females reported significantly higher ITN use than males in Wave 1 (52% *vs* 46%, p=0.017) and Wave 2 (62% *vs* 51%, p<0.0001). Similar patterns showing high female ITN use were observed by place of residence with higher ITN use among females compared to males in both urban (56% *vs* 44% p<0.0001) and rural (58% *vs* 52%, p=0.004) environments. Furthermore, females had significantly higher use of ITNs compared to males when analysed by wealth quintiles, particularly in the lowest (57% *vs* 46%, p=0.012) and fourth (58% *vs* 46%, p=0.003) quintiles. When the ratio of one ITN per two persons in a household was met, net use was equally high among women and men. In contrast, when the ratio was not met, females used nets more than the males (53% *vs* 48%, p<0.001). Age was also associated with ITN use. Among individuals below 15 years, the use of ITN was similar among females and males; however, at the age of 15 years and above, use of ITN was higher among females compared to males (Table 2).

**Table 2:** ITN use among individuals with access to ITNs, by gender and background characteristics

**Background characteristics**	**Female**		**Male**		**Total**		
	**Percentage**	**n**	**Percentage**	**n**	**Percentage**	**n**	P value*
**Total**	**57.2**	**1,562**	**48.8**	**1,494**	**53.1**	**3,056**	**<0.001**
**Campaign waves**							
Wave 1	52.4	391	46.2	344	49.3	1,490	**0.017**
Wave 2	61.5	502	51.3	385	56.6	1,566	**<0.001**
**Place of residence**							
Urban	55.6	306	43.8	232	49.8	1,080	**<0.001**
Rural	58.0	587	51.6	497	54.9	1,084	**0.004**
**Wealth quintiles**							
Lowest	57.4	162	46.4	111	52.4	521	**0.012**
Second	53.1	173	47.2	134	50.3	610	0.147
Middle	59.7	187	52.6	172	56.1	640	0.069
Fourth	58.0	193	46.3	148	52.2	653	**0.003**
Highest	57.8	178	50.6	164	54.1	632	0.070
**Ratio of 1 ITN/2 persons met**							
Yes	67.8	299	70.9	246	69.2	788	0.351
No	53.0	594	42.1	483	47.5	2268	**<0.001**
**Age**							
*Under 5 years*	62.4	303	61.9	336	62.1	639	0.902
*5-15 years*	49.0	498	48.0	473	48.5	971	0.754
*15-25 years*	58.1	270	23.4	197	43.5	467	**<0.001**
*25 years and older*	61.7	491	50.8	488	56.3	979	**0.001**

Bold =Significance assessed at 5% - comparing females and males.

### Sex as a predictor for ITN use

The results of the logistic regression show that females are more likely to use ITNs compared to males after controlling for a number of potential confounders, including: household wealth quintiles, campaign wave, age, place of residence, education of the head of household, polygamous status, and ratio of ITNs to household members (OR: 1.5, 95% CI: 1.3-1.7).

Among the covariates included in the model, the following were significantly associated with ITN use: campaign wave, age, education and ratio of ITNs to household members. After Wave 2 (July 2009), individuals were more likely to use ITNs compared to Wave 1 – May 2009 (OR: 1.4, 95% CI: 1.2-1.6). Age shows a significant effect on ITN use. Children under five years had higher odds of using an ITN compared to individuals aged 25 years or older (OR 1.4, 95% CI 1.2-1.6). Individuals aged 15–25 years were significantly less likely to use an ITN compared to individuals aged 25 years or older (OR: 0.6, 95% CI: 0.5-0.7). Education level of the head of household was associated with ITN use: in households with a head that had no formal education, ITN use was lower. The number of ITNs in the household was a strong predictor of ITN use; individuals living in households with at least one ITN for every two members were 2.5 times more likely to use an ITN compared to individuals living in households with less than one ITN for every two members. Such background characteristics as household wealth, place of residence, and polygamous households were not associated with ITN use (Table 3).

**Table 3:** Gender effects on the use of ITNs among individuals who have access to ITNs

**Factors**		**Number of individuals**	**Number of individuals using ITN (%)**	**Odd ratios (95% CI)**	***p value***
**Total number of individuals**		**3,056**	**1622 (53.1)**		
**Explanatory variable**					
Gender					
	*Male*	1,494	729 (48.8)	1	
	*Female*	1,562	893 (57.2)	**1.46 (1.25-1.70)**	**<0.001**
**Covariates**					
Wealth quintiles					
	*Lowest*	521	273 (52.4)	0.80 (0.61-1.05)	0.104
	*Second*	610	307 (50.3)	0.82 (0.64-1.06)	0.128
	*Middle*	640	359 (56.1)	1.09 (0.86-1.39)	0.479
	*Fourth*	653	341 (52.2)	0.89 (0.70-1.13)	0.334
	*Highest*	632	342 (54.1)	1	
Waves					
	*Wave 1*	1,490	735 (49.3)	1	
	*Wave 2*	1,566	887 (56.6)	**1.38 (1.18-1.61)**	**<0.001**
Age					
	*Under 5 years*	639	397 (62.1)	**1.41 (1.14-1.75)**	**0.002**
	*5-15 years*	971	471 (48.5)	0.88 (0.73-1.06)	0.169
	*15-25 years*	467	203 (43.5)	**0.57 (0.45-0.73)**	<0.001
	*25 years and older*	979	551 (56.3)	1	
Place of residence					
	*Urban*	1080	538 (49.8)	1	
	*Rural*	1976	1,084 (54.9)	1.15 (0.98-1.35)	0.098
Education of head of household					
	*None*	1,880	965 (51.3)	1	
	*Primary*	636	374 (58.8)	**1.38 (1.13-1.67)**	**0.001**
	*Secondary*	382	185 (48.4)	0.87 (0.68-1.12)	0.288
	*Higher*	115	75 (65.2)	**1.80 (1.16-2.78)**	**0.009**
	*Missing*	43			
Polygamous household					
	Yes	1,291	666 (51.6)	1	
	No	1,765	956 (54.2)	0.88 (0.76-1.03)	0.114
Ratio 1 ITN/2 persons met					
	*No*	2,268	1,077 (47.5)	1	
	*Yes*	788	545 (69.2)	**2.53 (2.11-3.04)**	**<0.001**

Model fit: LR chi2(18)=238.45; p value<0.00001; Pseudo R2=0.057; Log likelihood = −1963.54.

## Discussion

Using data on ITN use from the Kano State free long-lasting insecticidal net (LLIN) distribution post-campaign survey, the authors assessed the differences in ITN use by sex and other background characteristics, through a gender lens. The findings show that among all the individuals who lived in households with at least one ITN, females were more likely to use ITNs compared to males after controlling for several potential confounders. Although the campaign was not specifically designed to ensure equal access to ITNs or equal use between females and males, the concept of universal coverage implicitly attempts to promote equitable access to ITNs by all the different population groups. These findings call for a qualitative study to answer why males, in particular, are not accessing and using ITNs at similar levels, but, nonetheless, these results already deserve some attention to inform future campaigns as they attempt to reach universal coverage.

One possible reason for higher uptake of ITN among females over males is that pregnant women and children under five are considered the most vulnerable to malaria, influencing the nature of messages about mosquito net usage [8-10,14]. Billboard images, radio advertisements and television commercials often portray women and young children using nets, but, in general, media do not include men. It is possible that the universal campaign that was evaluated concentrated more on distributing LLINs than on providing messages about the importance of ITN use for everyone. A more in-depth analysis of messages from targeted campaigns *versus* mass campaigns would help to determine whether the messages being disseminated are gender neutral and whether they are accepted as such. It is essential that behaviour change messages take into account gender roles and norms and are consistent with common local practices.

The information above is also consistent with the findings related to the ratio of ITNs to the number of household members. If the overall use of ITNs is higher when this ratio is higher, then the converse should hold true: having fewer ITNs, and taking into account the traditional messaging on ITN use by women and children under five, men give priority to females when there are not enough ITNs in a household. This would be a rational decision because women often sleep under the same net with children under five, who are the most vulnerable. This explanation contradicts some suggestions that give the decision-making power in favour of males in the household and believe males will use the ITN if there are not enough for all household members [15,16]. More investigation into understanding household decision-making regarding ITN use in households that do not have sufficient ITNs to cover all members could elucidate the reasons for the observed differences in ITN use.

Another possible reason to explain why the women in the study used nets more than the men could be because women are less likely, in general, to take risks when it comes to health and are more likely, generally speaking, to adhere to health interventions and respond to health promotion messages. This has been observed in other health programmes, such as antiretroviral therapy (ART) compliance for HIV/AIDS and anti-smoking campaigns [17-19]. Knowledge that failure to use an ITN creates a health risk may be enough reason for a woman to use a net, particularly if one is available [20]. In contrast, men are more likely to be risk takers and would need more than just the knowledge of the risk to change behaviour [18,21,22]. Gender-specific behaviour change strategies could help to promote gender equity in ITN use as universal campaigns roll out across countries worldwide and may be needed to compensate for the historical behaviour change strategies which have primarily focused on women and children. While this study did not include collection of this type of data, it would be helpful to include such variables in future studies.

It would also be helpful to look at subgroups within the sexes when putting together a behaviour change strategy for a universal campaign. In this study, adolescent boys were least likely to use an ITN. Women with more wealth tended to use ITNs more than those without. Urban populations, both male and female, showed less ITN use than rural populations. Studying this information and using it to adjust a universal campaign strategy is essential to improving malaria control in a country and reducing malaria deaths. The results of this study do provide insightful information on the sex-specific outcomes of the free LLIN distribution campaign in Kano state, Nigeria. It is evident that these campaigns substantially increased ITN ownership and use among the general population and reduced the gap in ownership and use between different socioeconomic groups [7]. However, the observed gender disparity in ITN use is cause for concern as better population-level protection against malaria is a key objective of the universal coverage strategy.

This study would not be complete without mentioning a number of notable limitations. First, the post-campaign evaluation was not specifically designed to assess gender differences in ITN use. Although the sample size provided enough statistical power to measure sex differentials in ITN coverage and use, there was not sufficient information on social norms and determinants related to gender to fully interpret the results observed. Additional information on household decision-making would have been very informative.

Second, it should be noted that baseline data showed very low net coverage, so it was difficult to assess the situation prior to the campaign. Such information would have helped to better evaluate the effects of the campaign on gender differences in ITN use.

In summary, free ITN distribution campaigns are effective strategies to increase equitable access to ITNs for the general population; however, these campaigns may not always ensure equality in use among subpopulations. This study reveals gender disparity in ITN use in favour of females, i e, uptake of the intervention among females (traditionally considered the most at-risk group) is higher than among males. Further research is needed to identify whether gender disparity in ITN use is related to the lack of equity in access due to traditional targeting of pregnant women with malaria interventions or to the specific gender norms associated with male health-seeking behaviours. Nonetheless, results provide enough evidence to advocate for the design of gender-sensitive awareness messaging for ITN distribution to ensure that males, particularly the age group 15–25 years, benefit equally to the access and use of ITNs.

## Competing interests

The authors declare that they have no competing interests.

## Authors’ contributions

AG and YY conceived the paper, performed the analysis, and wrote the first draft of the manuscript. EI, EE, and SN contributed substantially to writing and reviewing the manuscript. All authors read and approved the final manuscript.
